# WHO Grade I Meningioma Recurrence: Identifying High Risk Patients Using Histopathological Features and the MIB-1 Index

**DOI:** 10.3389/fonc.2020.01522

**Published:** 2020-08-28

**Authors:** Alexander F. Haddad, Jacob S. Young, Ishan Kanungo, Sweta Sudhir, Jia-Shu Chen, David R. Raleigh, Stephen T. Magill, Michael W. McDermott, Manish K. Aghi

**Affiliations:** ^1^Department of Neurological Surgery, University of California, San Francisco, San Francisco, CA, United States; ^2^Department of Radiation Oncology, University of California, San Francisco, San Francisco, CA, United States; ^3^Miami Neuroscience Institute, South Miami, FL, United States

**Keywords:** meningioma, WHO grade I, recurrence, MIB-1, benign, early recurrence, late recurrence

## Abstract

**Objective:** In this study, we identify clinical, radiographic, and histopathologic prognosticators of overall, early, and post-median recurrence in World Health Organization (WHO) grade I meningiomas. We also determine a clinically relevant cutoff for MIB-1 to identify patients at high risk for recurrence.

**Method:** A retrospective review of WHO grade I meningioma patients with available MIB-1 index data who underwent treatment at our institution from 2007 to 2017 was performed. Univariate and multivariate analyses, and recursive partitioning analysis (RPA), were used to identify risk factors for overall, early (within 24 months), and post-median (>24 months post-treatment) recurrence.

**Result:** A total of 239 patients were included. The mean age was 60.0 years, and 69.5% of patients were female. The average follow-up was 41.1 months. All patients received surgery and 2 patients each received either adjuvant radiotherapy (2/239) or gamma knife treatment (2/239). The incidence of recurrence was 10.9% (26/239 patients), with an average time to recurrence of 33.2 months (6–105 months). Posterior fossa tumor location (*p* = 0.004), MIB-1 staining (*p* = 0.008), nuclear atypia (*p* = 0.003), and STR (*p* < 0.001) were independently associated with an increased risk of recurrence on cox-regression analysis. RPA for overall recurrence highlighted extent of resection, and after gross total resection (GTR), a MIB-1 index cutoff of 4.5% as key prognostic factors for recurrence. Patients with a GTR and MIB-1 >4.5% had a similar incidence of recurrence as those with STR (18.8 vs. 18.6%). Variables independently associated with early recurrence on binary logistic regression modeling included STR (*p* = 0.002) and nuclear atypia (*p* = 0.019). RPA confirmed STR as associated with early recurrence.

**Conclusion:** STR, posterior fossa location, nuclear atypia, and elevated MIB-1 index are prognostic factors for WHO grade I meningioma recurrence. Moreover, MIB-1 index >4.5% is prognostic for recurrence in patients with GTR. Verification of our findings in larger, multi-institutional studies could enable risk stratification and recommendations for adjuvant radiotherapy following resection of WHO grade I meningiomas.

## Introduction

Meningiomas are the most common primary central nervous system (CNS) neoplasm and account for over 37% of all primary brain tumors (1). Management options for meningiomas include observation, surgery, and radiotherapy (2, 3). While a minority of meningiomas are aggressive, including World Health Organization (WHO) grades II and III, over 80% are WHO grade I, and often called “benign” (4). However, even WHO grade I meningiomas can recur, with previous studies highlighting a recurrence rate of up to 47% with long-term follow-up (4, 5). Meningioma recurrence frequently necessitates treatment with additional surgery or salvage radiotherapy, leading to potential morbidity (6, 7). As a result, the ability to predict recurrence is a crucial component of WHO grade I meningioma management to make recommendations regarding the frequency of surveillance imaging, or the use of adjuvant radiotherapy.

In addition to the Simpson grade achieved at resection, a number of tumor characteristics have been evaluated as possible predictors of recurrence, with a focus on histopathological findings (8). These include the MIB-1 index (a marker of cell proliferation), brain invasion, and the presence of atypical histologic features, including increased cellularity, sheeting, foci of spontaneous necrosis, and nuclear atypia (9–13). Indeed, the 2016 WHO classifications exclusively use pathological findings to determine tumor grade with grade II defined by brain invasion and increased mitosis over 4/10 high powered field (HPF) (4). In addition, three atypical features together result in an increased tumor grade (4). Looking forward, meningioma molecular characteristics have recently been associated with risk of recurrence, and will likely be used in meningioma grading in the future (14–16).

While the WHO classifications synthesize the available literature to create clear delineators between tumor grades, the literature surrounding WHO grade I meningioma recurrence remains mixed. For example, while brain invasion alone can result in an increase of tumor grade from WHO grade I–II, a number of subsequent studies have not found a relationship between brain invasion and recurrence, highlighting the need for additional research into predictors of recurrence (10, 17, 18). The utility of the MIB-1 index in predicting meningioma recurrence is also controversial; a study in WHO grade I meningiomas only suggested a higher recurrence risk with a MIB-1 index of >3%, but other literature including all WHO grades have demonstrated a higher incidence of recurrence at >5% or >10% (9–11). Previous studies have also proposed a MIB-1 cutoff of >3% only in patients with a Simpson II or III resection, further demonstrating the variety in MIB-1 cutoffs and their usage (13). In comparison, a limited number of studies have investigated the impact of atypical features in WHO grade I meningioma recurrence; although, they have suggested a higher risk of recurrence in WHO grade I tumors displaying atypical features upon pathologic analysis (9).

Therefore, the goal of this study was to investigate clinical, radiographic, and pathologic predictors of recurrence in WHO grade I meningiomas. Given the lack of consensus on the use of MIB-1 in WHO grade I meningiomas, we also utilize recursive partitioning analysis to identify a clinically relevant cutoff for the MIB-1 index. We then identify predictors of early and post-median recurrence in WHO grade I tumors.

## Methods

### Patient Population

Patients who underwent treatment for a WHO grade I meningioma from 2007 to 2017 were retrospectively identified using an institutional database. This study was formally approved by the University of California, San Francisco Institutional Review Board (IRB#13-12587). Patients without MIB-1 index values available in the electronic medical record were excluded. Early in the study period, MIB-1 was obtained at the discretion of the attending neuropathologist, but, as time progressed, MIB-1 was obtained on all WHO grade I meningioma patients.

### Clinical Data

Patient demographics, clinical, and treatment characteristics, histopathological data, and clinical outcomes were retrospectively reviewed and collected. Clinical data collected included patient age, sex, Karnofsky Performance Status (KPS) at treatment, clinical presentation, and previous history of meningioma treatment. Histopathologic data included MIB-1 index, sheeting/loss of architecture, increased cellularity, necrosis, nuclear atypia, and the presence of bone invasion. Progesterone receptor (PR), Epithelial Membrane Antigen (EMA), CD34, S100, glial fibrillary, and acidic protein (GFAP) staining results were collected when available. Pathologic data was extracted from the pathology report generated at the time of surgical intervention. Tumor location, size, and the presence of preoperative peritumoral edema were determined using preoperative MRI imaging. Anterior-posterior (AP), superior-inferior (SI), and transverse (TV) diameters were collected. Preoperative tumor volume was calculated using the equation for non-spherical tumor volume. Treatment type was similarly collected. Simpson grade was determined through the operative report. Gross total (GTR) and Subtotal resection (STR) were determined using post-operative magnetic resonance imaging (MRI) scans.

The primary outcomes of interest were tumor recurrence and time to recurrence. Recurrence was determined on post-operative radiography as a local recurrence or progression of residual tumor. Time to recurrence was determined from the patient's date of treatment. Length of follow-up was calculated from the date of treatment to the last visit with the neurosurgery clinic. Secondary outcomes included early and post-median recurrence. Early recurrence was defined as within 2 years of initial treatment (19). Post-median recurrence was defined as occurring >2 years [based on previous literature (19) and the median time to recurrence in the cohort] following initial treatment.

### Statistical Analysis

Chi-square and Student's *t*-test were utilized for the comparison of categorical and continuous variables, respectively. A multivariate backward likelihood Cox regression model for recurrence was constructed using variables with *p* < 0.200 on univariate analysis. Similarly, multivariate backward likelihood binary logistic models were constructed to predict early and post-median recurrence using variables with *p* < 0.200 on univariate analysis. Recursive partitioning analysis (RPA) was used to further identify key risk factors of overall, early, and post-median meningioma recurrence. A *p* < 0.050 was used as a threshold of statistical significance. All statistical analysis was performed with SPSS 26.

## Results

### Overall Patient Demographics and Clinical Outcomes

Overall patient demographics, histopathological features, and clinical outcomes can be seen in Table 1. In total, 239 patients with WHO grade I meningiomas were included in the study. The average age was 60.0 years, and 69.5% of patients were female. The most common presenting symptom was headache (30.5%), followed by a focal neurologic deficit (27.6%). The majority of patients underwent surgery alone (98.3%), with 2 patients each receiving adjuvant radiation therapy (0.8%) or gamma knife (0.8%) treatments. Peritumoral edema was present on the preoperative MRIs of 38.1% of patients. The average calculated tumor volume was 30.6 cm^3^ (range = 0.23–215.73 cm^3^), and the average largest tumor dimension was 3.8 cm (range = 0.6–11.3 cm). Overall, 91 (38.1%) tumors were located on the skull base, 57 (23.8%) were convexity tumors, 43 (18.0%) had a falx/parasagittal location, and 55 (23.0%) had another location. Most patients received a gross total resection (63.6%). The incidence of Simpson I, II, III, and IV resection were 31.4, 29.7, 2.5, and 35.6%, respectively. Atypical features were present in a number of patients; the most common atypical features were bone invasion (18.0%) and sheeting/loss of architecture (8.4%). Mean follow-up was 41.1 months (range: 0–147 months).

**Table 1:** Patient characteristics, histopathological features, radiography, and outcomes.

**Variable**	***N* = 239**
**Age (years)**	
Mean (range)	60.0 (27–90)
Number of female patients	166 (69.5%)
**Follow-up**	
Mean (range)	41.1 (0–147)
**Presenting symptoms**	
Headache	73 (30.5%)
Seizure	35 (14.6%)
Cognitive changes	23 (9.6%)
Focal neurologic deficit	66 (27.6%)
Extremity weakness	28 (11.7%)
Ataxia	14 (5.9%)
Vertigo	25 (10.5%)
Proptosis	9 (3.8%)
None (incidental)	53 (22.2%)
KPS at treatment (*N* = 237)	84.7 (30–100)
**Tumor location**	
Falx/parasagittal	43 (18.0%)
Convexity	57 (23.8%)
Skull base	91 (38.1%)
Posterior fossa	18 (7.5%)
Middle fossa	55 (23.0%)
Anterior fossa	22 (9.2%)
Other	55 (23.0%)
Recurrent tumor	7 (2.9%)
**Radiographic characteristics**	
Peritumoral edema	91 (38.1%)
Tumor volume (*N* = 164) (range)	30.6 (0.23–215.73)
Largest tumor dimension (*N* = 199) (range)	3.8 (0.6–11.3)
**Histopathology**	
MIB-1 (average)	3.3 (0.0–18.11)
Sheeting/loss of architecture	20 (8.4%)
Increased cellularity	16 (6.7%)
Necrosis	24 (10.0%)
Nuclear atypia	21 (8.8%)
Bone invasion	43 (18.0%)
**Treatment characteristics**	
Surgery	235 (98.3%)
Surgery + radiation therapy	2 (0.8%)
Surgery + gamma knife	2 (0.8%)
Preoperative embolization	41 (17.2%)
**Simpson grade**	
I	76 (31.8%)
II	71 (29.7%)
III	6 (2.5%)
IV	86 (36.0%)
**EOR**	
STR	86 (36.0%)
GTR	153 (64.0%)
Recurrence	26 (10.9%)
Mean months to recurrence	33.2 ± 23.7
Median months to recurrence (range)	24.5 (6–105)
Multiple recurrence	5 (2.1%)

### Predictors of WHO Grade I Meningioma Recurrence

A total of 26 patients recurred with a median time to recurrence of 24.5 months (Table 1). A comparison between patients with a recurrence and those without can be found in Table 2. There was no difference in age (60.2 vs. 58.8, *p* = 0.582) or female gender (61.5 vs. 70.4%, *p* = 0.353) between patients with tumors that recurred and non-recurrent patients. Treatment characteristics were similar between the groups, with most patients in each group receiving surgery alone (100.0 vs. 98.1%, *p* = 1.000). However, patients with recurrent tumors had a higher incidence of STR (61.5 vs. 32.7%, *p* = 0.004) and a lower incidence of Simpson grade I resection (11.5 vs. 34.1%, *p* = 0.019). The incidence of peritumoral edema, tumor size, and tumor volume were similar between the groups. On histopathologic analysis, patients with recurrence trended toward increased nuclear atypia (19.2 vs. 7.5%, *p* = 0.061). Patients with recurrence also had a higher mean follow-up (68.9 vs. 37.7 months, *p* = 0.001).

**Table 2:** Comparison of patients with recurrent meningiomas vs. non-recurrent meningiomas.

**Variable**	**Non-recurrent**	**Recurrent**	***P*-value**
	***N* = 213**	***N* = 26**	
**Age (years)**			
Mean	58.81 ± 12.30	60.23 ± 13.02	0.582
Number of female patients	150 (70.4%)	16 (61.5%)	0.353
**Follow-up (months)**			
Mean (range)	37.72 ± 30.62	68.85 ± 42.25	0.001
KPS at treatment	86.02 ± 11.72	85.77 ± 10.27	0.917
**Tumor location**			
Falx/parasagittal	38 (17.8%)	5 (19.2%)	0.792
Convexity	53 (24.9%)	4 (15.4%)	0.283
Skull base	80 (37.6%)	11 (42.3%)	0.638
Posterior fossa	14 (6.6%)	4 (15.4%)	0.108
Middle fossa	52 (24.4%)	3 (11.5%)	0.141
Anterior fossa	18 (8.5%)	4 (15.4%)	0.274
Other	49 (23.0%)	6 (23.1%)	0.993
Previously treated tumor	5 (2.3%)	2 (7.7%)	0.127
**Radiographic characteristics**			
Peritumoral edema	82 (38.5%)	9 (34.6%)	0.700
Tumor volume (*N* = 164)	30.48 ± 36.21	32.61 ± 38.79	0.834
•Largest tumor dimension (*N* = 239)	3.22 ± 2.03	2.8 ± 2.58	0.358
**Histopathology**			
MIB-1 (%)	3.22 ± 2.23	4.27 ± 3.70	0.168
•Sheeting/loss of architecture	17 (8.0%)	2 (11.5%)	0.464
Necrosis	20 (9.4%)	4 (15.4%)	0.309
Increased cellularity	13 (6.1%)	3 (11.5%)	0.295
Nuclear atypia	16 (7.5%)	5 (19.2%)	0.061
Bone invasion	36 (16.9%)	7 (26.9%)	0.209
**Treatment characteristics**			
Surgery	209 (98.1%)	26 (100.0%)	1.000
Preoperative embolization	36 (16.9%)	5 (19.2%)	0.784
**Simpson grade**			
I	73 (34.3%)	3 (11.5%)	0.019
II	65 (30.8%)	6 (23.1%)	0.417
III	5 (2.4%)	1 (3.8%)	0.506
IV	70 (32.9%)	16 (61.5%)	0.004
**EOR**			0.004
STR	70 (32.9%)	16 (61.5%)	
GTR	143 (67.1%)	10 (38.5%)	

**Figure 1:**
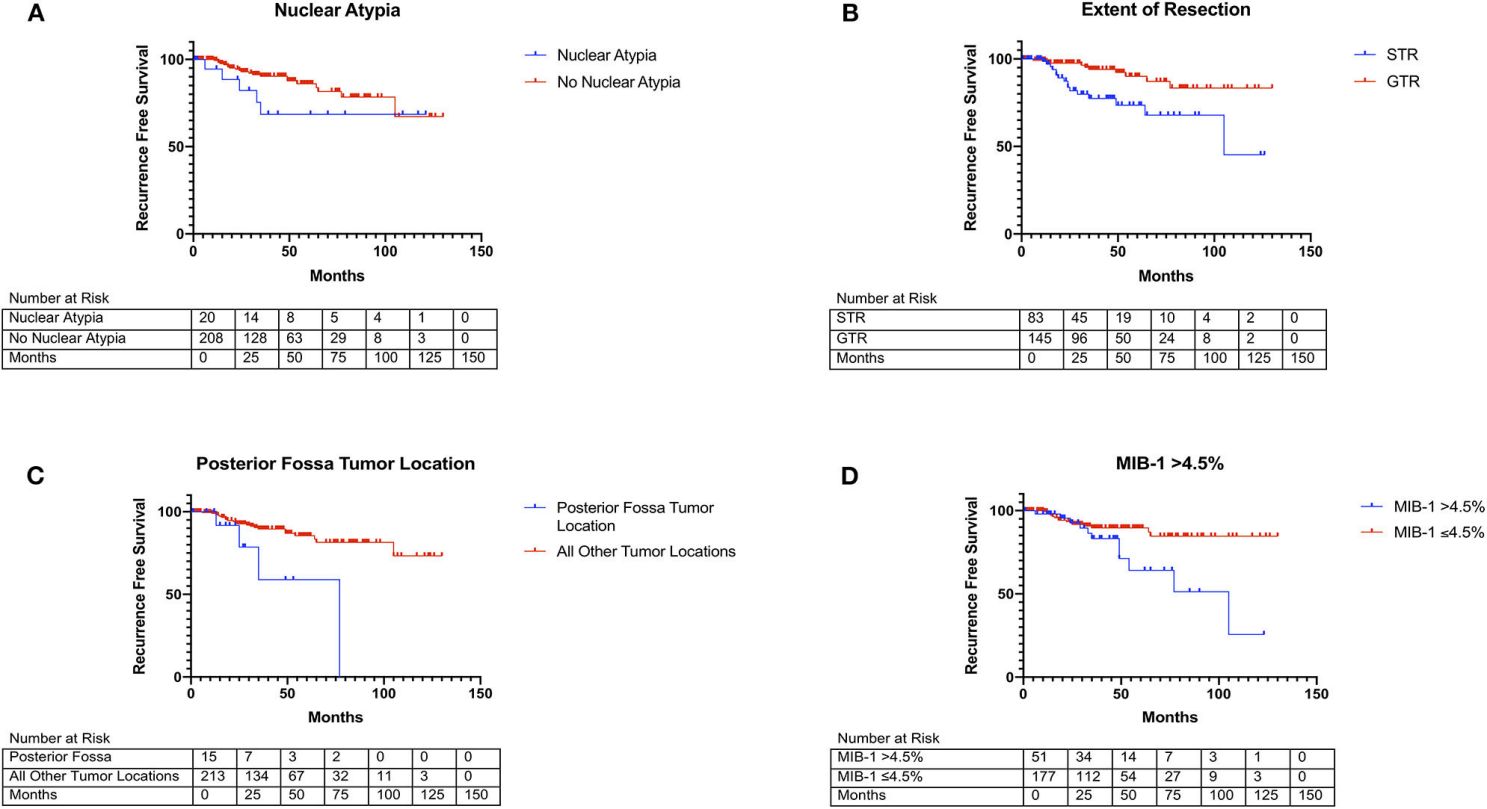
Kaplan Meier curves of risk factors for WHO grade I meningioma recurrence. **(A)** Nuclear atypia (blue line) vs. no nuclear atypia (red line) (*X*^2^ = 2.21, *p* = 0.137). **(B)** Posterior-fossa tumor location (blue line) vs. other locations (red line) (*X*^2^ = 10.36, *p* = 0.001). **(C)** MIB-1 index >4.5% (blue line) vs. ≤4.5% (red line) (*X*^2^ = 6.17, *p* = 0.013). **(D)** STR (blue line) vs. GTR (red line) (*X*^2^ = 10.46, *p* = 0.001).

A subsequent backward likelihood cox-regression analysis highlighted an independent relationship between recurrence and posterior fossa tumor location (HR = 5.25, CI 1.71–16.17, *p* = 0.004), MIB-1 index (HR = 1.18, CI 1.05–1.34, *p* = 0.008), nuclear atypia (HR = 5.24, CI 1.73–15.92, *p* = 0.003), and STR (HR = 5.66, CI 1.30–13.92, *p* < 0.001; Table 3). Kaplan-Meier curves highlighting recurrence free survival for posterior fossa location (*p* = 0.007), nuclear atypia (*p* = 0.137), extent of resection (*p* = 0.001), and MIB-1 >4.5% (*p* = 0.001) are shown in Figures 1A–D.

**Table 3:** Cox regression analysis for recurrence.

**Variable**	**Hazard ratio**	**95% CI**	***P*-value**
**Tumor location**			
All other tumor locations	Ref		
Middle fossa location	0.326	0.093–1.14	0.080
Posterior fossa location	5.27	1.71–16.20	0.004
MIB-1 staining	1.18	1.05–1.34	0.008
**Nuclear atypia**			
No nuclear atypia	Ref		
Nuclear atypia	5.26	1.73–15.97	0.003
**Extent of resection**			
Gross total resection	Ref		
Subtotal resection	5.68	2.31–13.97	<0.001

### Predictors of Early vs. Post-median WHO Grade I Meningioma Recurrence

A comparison between non-recurrent patients and patients with tumors that recurred early or post-median is presented in Table 4. Patients with an early recurrence had a higher incidence of subtotal resection (76.9 vs. 32.7%, *p* = 0.001). They also trended toward a higher incidence of nuclear atypia (23.1 vs. 7.5%, *p* = 0.084) on histopathology (Table 4). Patients with a post-median recurrence trended toward an increased incidence of posterior fossa tumor location (23.1 vs. 6.6%, *p* = 0.063) and a higher incidence of bone invasion (38.5 vs. 16.9%, *p* = 0.064) as well as a higher MIB-1 index (5.55 vs. 3.22%, *p* = 0.098). There was no significant difference between Simpson grading or GTR rates in patients with post-median recurrence vs. non-recurrent patients (Table 4).

**Table 4:** Early vs. post-median recurrence.

**Variable**	**Non-recurrent**	**Early**	***P*-value (vs**.	**Post-median**	***P*-value (vs**.
	***N* = 213**	***N* = 13**	**non-recurrent)**	***N* = 13**	**non-recurrent)**
**Age (years)**					
Mean	58.81 ± 12.30	57.00 ± 13.98	0.609	63.46 ± 11.63	0.186
Number of female patients	150 (70.4%)	9 (69.2%)	1.000	7 (53.8%)	0.224
**Follow-up (months)**					
Mean (range)	37.72 ± 30.62	55.92 ± 39.24	0.125	81.77 ± 42.63	0.003
**Tumor location**					
Falx/parasagittal	38 (17.8%)	4 (30.8%)	0.269	1 (7.7%)	0.703
Convexity	53 (24.9%)	1 (7.7%)	0.311	3 (23.1%)	1.000
Skull base	80 (37.6%)	4 (30.8%)	0.772	7 (53.8%)	0.241
Posterior fossa	14 (6.6%)	1 (7.7%)	0.601	3 (23.1%)	0.063
Middle fossa	52 (24.4%)	1 (7.7%)	0.309	2 (15.4%)	0.738
Anterior fossa	18 (8.5%)	3 (23.1%)	0.108	1 (7.7%)	1.000
Other	49 (23.0%)	4 (30.8%)	0.509	2 (15.4%)	0.738
Recurrent tumor	5 (2.3%)	1 (7.7%)	0.302	1 (7.7%)	0.302
**Radiographic characteristics**					
Peritumoral edema	82 (38.5%)	7 (55.8%)	0.272	2 (15.4%)	0.139
Tumor volume (*N* = 158) (*N* = 156)	30.48 ± 36.21	26.03 ± 22.17	0.732	41.38 ± 55.34	0.480
Largest tumor dimension (*N* = 226)	3.22 ± 2.03	2.39 ± 2.14	0.157	3.25 ± 2.99	0.978
**Histopathology**					
MIB-1	3.22 ± 2.23	2.99 ± 1.82	0.722	5.55 ± 4.65	0.098
Sheeting/loss of architecture	17 (8.0%)	2 (15.4%)	0.300	1 (7.7%)	1.000
Necrosis	20 (9.4%)	1 (7.7%)	1.000	3 (23.1%)	0.134
Increased cellularity	13 (6.1%)	1 (7.7%)	0.575	2 (15.4%)	0.210
Nuclear atypia	16 (7.5%)	3 (23.1%)	0.084	2 (15.4%)	0.277
Bone invasion	36 (16.9%)	2 (15.4%)	1.000	5 (38.5%)	0.064
**Treatment characteristics**					
Surgery	209 (98.1%)	13 (100.0%)	1.000	13 (100.0%)	1.000
Preoperative embolization	36 (16.9%)	2 (15.4%)	1.000	3 (23.1%)	0.474
**Simpson grade (*****N*** **= 224)**					
I	73 (34.3%)	1 (7.7%)	0.065	2 (15.4%)	0.229
II	65 (30.8%)	2 (15.4%)	0.353	4 (30.8%)	1.000
III	5 (2.4%)	0 (0.0%)	1.000	1 (7.7%)	0.304
IV	70 (32.9%)	10 (76.9%)	0.002	6 (46.2%)	0.370
**EOR**			0.001		0.370
STR	70 (32.9%)	10 (76.9%)		6 (46.2%)	
GTR	143 (67.1%)	3 (23.1%)		7 (53.8%)	
**Time to recurrence**					
Mean (range)		16.4 ± 5.2	<0.001^*^	50.1 ± 23.0	<0.001^*^

* *Post-median vs. early recurrence*.

Two multivariate backward likelihood binary logistic models were used to identify predictors of early vs. post-median recurrence (Table 5). Independent predictors of early tumor recurrence included nuclear atypia on histopathology (OR = 6.45, CI 1.34–31.07, *p* = 0.020) and STR (OR = 8.92, CI 2.18–36.46, *p* = 0.002). The sole independent predictor of post-median recurrence was MIB-1 index (OR = 1.24, CI 1.05–1.45, *p* = 0.010), although posterior fossa location approached significance (OR = 4.42, CI 0.954–20.49, *p* = 0.058; Table 5).

**Table 5:** Binary logistic model for post-median and early tumor recurrence.

	**Variable**	**Odds ratio**	**95% CI**	***P*-value**
Post-median tumor recurrence	Tumor location			
	Non-posterior fossa location	Ref		
	Posterior fossa location	4.42	0.954–20.49	0.058
	MIB-1 staining	1.24	1.05–1.45	0.010
	Peritumoral edema			
	No peritumoral edema	Ref		
	Peritumoral edema present	0.266	0.055–1.29	0.101
Early tumor recurrence	Extent of resection			
	Gross total resection	Ref		
	Subtotal resection	8.87	2.17–36.28	0.002
	Nuclear atypia			
	No nuclear atypia	Ref		
	Nuclear atypia present	6.53	1.37–31.43	0.019

### Recursive Partitioning Analysis of Recurrence

**Figure 2:**
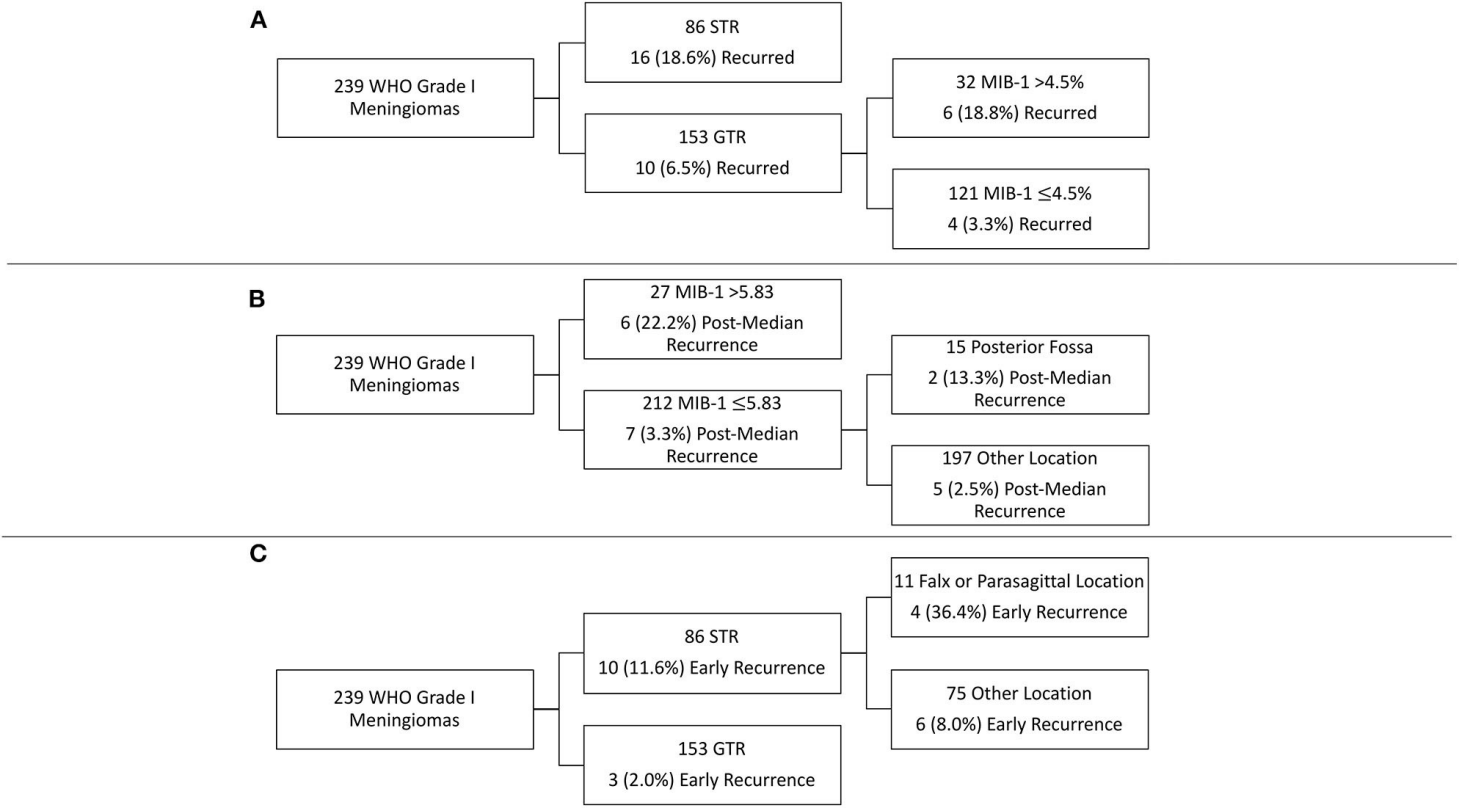
Recursive partitioning analysis highlighting key risk factors for **(A)** overall recurrence, **(B)** post-median recurrence, and **(C)** early recurrence.

RPA was performed to identify key risk factors of meningioma recurrence (Figure 2). Consistent with the cox-regression analysis, STR was the first partition when predicting overall recurrence: 18.6% of patients with an STR resection recurred as compared to 6.5% with a GTR. The next decision node within only GTR patients involved a MIB-1 cutoff of 4.5%, as 18.8% of patients with a MIB-1 >4.5% recurred vs. 3.3% of patients with a MIB-1 ≤4.5% (Figure 2A). With regards to post-median recurrence specifically, RPA identified the first decision node as a MIB-1 cutoff of 5.83%: 22.2% of patients with MIB-1 >5.83% recurred vs. 3.3% of patients with a MIB-1 ≤5.83%. The subsequent decision node utilized a posterior fossa tumor location: 13.3% of patients with a posterior fossa tumor recurred vs. 2.5% of other tumor locations (Figure 2B). RPA for early recurrence identified extent of resection as the primary decision node: 11.6% of patients with STR recurred vs. 2.0% of GTR patients. The following decision node utilized falx or parasagittal location: 36.4% of falx or parasagittal tumors recurred vs. 8.1% of other tumor locations (Figure 2C).

## Discussion

### Key Results

WHO grade I meningioma recurrence is independently associated with MIB-1 index, posterior fossa tumor location, the presence of nuclear atypia, and STR. More specifically, a MIB-1 index of 4.5% was identified as a clinically relevant cutoff in risk-stratifying WHO grade I meningioma patients following GTR. Patients with a >4.5% MIB-1 index and GTR of their WHO grade I meningioma had a similar risk of recurrence as those patients with an STR. Further analysis highlighted MIB-1 as a critical factor associated with post-median recurrence while extent of resection was the main driver of early recurrence.

### Predictors of WHO Grade I Meningioma Recurrence

Previous studies have similarly assessed the relationship between histopathological features, clinical characteristics, and recurrence in all meningioma WHO grades. In a study of 901 patients (716 WHO Grade I, 174 Grade II, and 11 Grade III), Gousias et al. demonstrate a higher risk of recurrence in meningiomas with a MIB-1 index of >10%, higher WHO grade, tumor size >6 cm, petroclival or cavernous sinus location, and multiplicity (11). However, Gousias et al. did not assess the relationship between the presence of atypical features on histology and tumor recurrence. In WHO grade I meningiomas specifically, Marciscano et al. utilized a cohort of 148 WHO grade I meningioma patients with complete pathological analysis to identify variables associated with recurrence, with a focus on the impact of atypical pathologic features on recurrence risk (9). Interestingly they identify the presence of atypical features as an independent risk factor in addition to MIB-1 index >3% and Simpson resection. Our study similarly highlights surgical GTR and nuclear atypia, an atypical feature, as independent predictors of recurrence (Table 3), although we assessed each atypical feature independently. We also consider tumor location in our cox-regression model, further identifying posterior fossa location of the tumor as an independent risk factor of recurrence. While Marciscano et al. did not include tumor location in their analysis of predictors of progression, Gousias et al. similarly demonstrate petroclival tumor location as a risk factor for recurrence, albeit when considering all WHO meningioma grades (9, 11). The higher risk of recurrence associated with posterior fossa location may be due to the increased prevalence of NF2 mutations in the posterior fossa (14, 15, 20), although we are unable to fully explore this as we do not routinely perform genetic testing of meningioma at our institution. Regardless, additional studies with larger WHO grade I meningioma patient cohorts are needed to investigate this relationship.

### MIB-1 Index and WHO Grade I Meningioma Recurrence

Similar to previous studies (9, 11, 13, 21) we then also evaluated the relationship between the MIB-1 index of a tumor and its risk of recurrence. We first evaluated MIB-1 index in a cox-regression model for overall recurrence, as previously discussed. In agreement with previous studies, we found that higher MIB-1 index was independently associated with an overall increased risk of recurrence. Given the discrepancies in cutoff values for MIB-1 between the literature and the inter-laboratory variability, we initially evaluated MIB-1 as a continuous variable. We next sought to determine the cutoff for MIB-1 index in our patient population by utilizing RPA to model overall recurrence, which identified a MIB-1 cutoff value of 4.5% in patients with GTR (Figure 2A). Interestingly, these patients had a similar risk of recurrence as patients with an STR, demonstrating the utility of MIB-1 in patients following GTR. Perry et al. similarly identifies a MIB-1 index of 4.2% as associated with recurrence following GTR on univariate analysis, albeit when considering all meningioma grades (22).

The recurrence rate of 18.8% in patients with a GTR and MIB-1 >4.5%, highlights the need for close surveillance of these patients or even the consideration of adjuvant radiation therapy, depending on patient preference. Adjuvant radiation therapy following resection for WHO grade I meningioma has been shown to reduce recurrence, especially following STR (23–25), although observation following STR remains standard practice (8). In a study of 92 WHO grade I meningiomas, Soyuer et al. demonstrated a 91% progression-free survival (PFS) in patients who received adjuvant radiotherapy following STR, which was significantly higher than the 38% PFS in those patients who had not received adjuvant radiotherapy (24). As patients in our study with an STR had a similar risk of recurrence as those with a GTR and MIB-1 >4.5%, adjuvant radiotherapy for both groups may reasonable. However, larger studies are needed to further validate our MIB-1 cutoff and the associated clinical implications. Prospective studies investigating the use of adjuvant radiotherapy in WHO grade I meningiomas are also needed.

### Predictors of Early and Post-median WHO Grade I Meningioma Recurrence

Given the trend toward a later recurrence in patients with an elevated MIB >4.5% (Figure 1C), we then sought to investigate differences in predictors between patients who recurred early (defined as within 2 years of treatment) or post-median (those who recurred >2 years following treatment). Few studies in the literature have investigated predictors of early recurrence in meningiomas. A study by Budohoski et al. identifies parafalcine location, STR, and peritumoral edema on radiographic imaging as predictors of early recurrence in a cohort of 220 atypical meningiomas (26). A similar study by Maillo et al., in WHO grade I meningiomas, utilizes interphase fluorescence *in situ* hybridization (FISH) and pathological features to identify risk factors for early recurrence (defined as 2.5 years after treatment) and found larger tumor size, karyotype abnormalities, patient age, and abnormalities of chromosome 10 to be associated with increased risk of recurrence (19). However, the authors did not consider the presence of atypia on pathology or MIB-1 index. Using a cohort of WHO grade I meningioma patients with more granular pathologic and clinical data, we identified extent of resection and nuclear atypia on pathology as independent predictors of early recurrence on binary logistic regression modeling (Table 5). RPA similarly revealed the importance of extent of resection in risk-stratifying patients for early recurrence (Figure 2C). Interestingly, it also highlighted the increased risk associated with falx/parasagittal tumor location, corresponding with the findings of Budohoski et al. (26). The only significant predictor of post-median tumor recurrence on binary logistic regression modeling was MIB-1 index, although posterior fossa location of the tumor approached significance. This was further demonstrated on RPA (Figure 2B). Surprisingly, extent of resection was not an independent predictor of post-median recurrence, as the majority of post-median recurrences had undergone a GTR. Post-median recurrences may highlight a category of WHO grade I meningioma that is molecularly more aggressive and recurs despite GTR, given their elevated MIB-1 index and posterior fossa location [potentially indicating an underlying NF2 mutation (20)]. Early recurrences are significantly more impacted by extent of resection and, as a result, likely represent the continued growth of residual tumor as opposed to the recurrence of previous completely resected tumor, as seen in post-median recurrences. Thus, our results potentially represent two different molecular WHO grade I meningioma subtypes. However, future studies with larger patient cohorts and molecular tumor data are needed to further investigate the underlying differences in molecular alterations between early and post-median recurrences.

### Limitations

The limitations of our study include its retrospective nature and low incidence of recurrence. Our study also includes patients beginning in 2007 and, as a result, spans both the 2007 and 2016 WHO CNS classification schemes. However, the only significant change between the two classifications was the addition of brain invasion as a lone criterion for WHO grade II status. Only two of the patients included in our cohort had brain invasion noted on pathological analysis, and neither patient recurred. As a result, we do not believe the inclusion of patients graded using the 2007 classification scheme had a significant impact on our findings. In addition, our study relies on the pathologic reports following initial resection, without a central re-review of pathologic slides. This may lead to increased variability in the pathologic variables, such as nuclear atypia and other similar pathologic findings which can demonstrate interobserver variability (27, 28). However, MIB-1 is a relatively objective measure with lower inter-observer variability between pathologists using the same method within a pathology laboratory (29). Practice patterns regarding MIB-1 testing also changed during the study time-period from individual pathologist preference, which varied depending on the pathologist, to testing in all patients. Factors considered by pathologists when deciding on MIB-1 testing included the atypical features included in our study, thus minimizing their potential impact on our findings. In addition, all meningiomas included in our study are WHO Grade I. As a result, we believe any bias introduced into the study based on these changes in practices patterns is minimal. Finally, while we include detailed clinical and pathologic characteristics, our patient cohort lacks information regarding tumor genetic and molecular changes, which have been shown to have a significant impact on tumor outcomes (15, 30–32). Thus, there remains a need for additional large multi-institutional studies with molecular/genetics data in addition to traditional pathologic and clinical variables when predicting overall recurrence of WHO grade I meningiomas. This includes consideration of molecular/genetic prognosticators, such as genome-wide methylation patterns (33, 34), TERT promoter mutations (35), and additional tumor molecular data given our findings, future investigations into unique genetic/molecular, clinical, and pathologic predictors of early and later recurrences are warranted as well, given the potential impact on therapeutic decision making by physicians.

Nevertheless, our study provides detailed insight into clinical and histopathological predictors of recurrence, specifically in WHO grade I meningiomas. In addition, we identify patients with a MIB-1 >4.5% as being at high risk for recurrence following GTR. We also leverage our data to provide insight into differences between early and post-median WHO grade I meningioma recurrences, potentially identifying different WHO grade I meningioma molecular subgroups. Our results suggest that patients with an elevated MIB-1 index and nuclear atypia on pathologic analysis, posterior fossa location of their tumor, and STR are at higher risk for recurrence and should be considered for closer follow-up or even adjuvant radiotherapy. In addition, patients with a MIB-1 over 4.5% are at a similar risk for recurrence as those who have undergone STR of their tumors and should also potentially be considered for adjuvant radiotherapy.

## Conclusion

There remains a paucity of literature on specific predictors of recurrence in WHO grade I meningiomas. The findings of this study highlight posterior fossa tumor location, MIB-1 index, nuclear atypia, and extent of resection as independently associated with recurrence of grade I meningiomas. We also demonstrate that patients with a MIB-1 >4.5% and GTR have a similar risk of recurrence as patients with an STR. Finally, differential analysis of early and post-median recurrences revealed the association of MIB-1 index and posterior fossa location in post-median recurrences, while early recurrences were more significantly impacted by extent of resection. Additional studies validating our findings and including molecular/genetic data are needed to identify additional predictors of recurrence in WHO grade I meningiomas. Such studies could provide a more accurate framework to risk-stratify patients and aid with therapeutic decision making, including the potential for adjuvant radiotherapy.

## Data Availability Statement

The raw data supporting the conclusions of this article will be made available by the authors, without undue reservation.

## Ethics Statement

The studies involving human participants were reviewed and approved by UCSF Institutional Review Board. Written informed consent for participation was not required for this study in accordance with the national legislation and the institutional requirements.

## Author Contributions

AH: conceptualization, methodology, formal analysis, investigation, writing—original draft, writing—review and editing, and visualization. JY: conceptualization, methodology, and writing—review and editing. IK and SS: investigation. J-SC: visualization and formal analysis. DR and MM: writing—review and editing. SM: writing—review and editing, conceptualization, and methodology. MA: writing—review and editing, and supervision. All authors contributed to the article and approved the submitted version.

## Conflict of Interest

The authors declare that the research was conducted in the absence of any commercial or financial relationships that could be construed as a potential conflict of interest. The reviewer BB declared a past co-authorship with the authors DR, MM, and SM to the handling editor.
